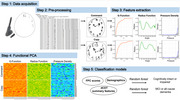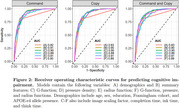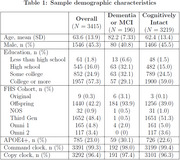# Predicting cognitive impairment using novel functional features of spatial proximity and circularity in the digital clock drawing test

**DOI:** 10.1002/alz70860_098476

**Published:** 2025-12-23

**Authors:** Adlin Pinheiro, Cody Karjadi, Yorghos Tripodis, Vijaya B. Kolachalama, Kathryn L. Lunetta, Serkalem Demissie, Chunyu Liu, Rhoda Au, Shariq Mohammed

**Affiliations:** ^1^ Boston University School of Public Health, Boston, MA, USA; ^2^ Framingham Heart Study, Boston, MA, USA; ^3^ Framingham Heart Study, Framingham, MA, USA; ^4^ Boston University Chobanian & Avedisian School of Medicine, Boston, MA, USA; ^5^ Section of Computational Biomedicine, Department of Medicine, Boston University School of Medicine, Boston, MA, USA; ^6^ Boston University, Boston, MA, USA; ^7^ The Framingham Heart Study, Framingham, MA, USA

## Abstract

**Background:**

The digital clock drawing test (dCDT) is a cognitive screening tool employing a digital pen to capture high‐resolution pen movements. Traditional dCDT approaches to predict cognitive outcomes often rely on many *summary features* (e.g. time to completion, mean pressure, clock face area, etc.) which involve subjective decisions such as feature selection and imputation of missing data. To address these limitations, we introduce novel dCDT features, expressed as mathematical functions. These *functional features* include the G‐function (measuring spatial proximity of points), pressure density function (measuring variability in pressure exerted on the paper), and radius function (measuring circularity of the clock face). We compare the performance of these functional features to 45 commonly used summary features.

**Methods:**

We included dCDTs from 3,415 stroke‐free participants from the Framingham Heart Study with at least one command or copy task. Random forest models with five‐fold cross‐validation were trained to distinguish participants with MCI or dementia from cognitively intact participants. Predictors included combinations of demographics, time‐based features, summary features, and functional features. Functional features underwent dimension reduction using functional principal components analysis. Area under the receiver operating curve (AUC), sensitivity, and specificity assessed model performance.

**Results:**

Average age was 63.6 years (SD: 13.9), with 1,546 (45%) males, 785 (23%) having at least one APOE‐e4 allele, and 1,957 (57%) having a college education. At the time of the dCDT, 122 (4%) had mild cognitive impairment and 74 (2%) had dementia. When combined with demographics and time‐based features, functional features from command and copy tests related to spatial proximity (AUC: 0.90; 95% CI: 0.88‐0.92) and circularity (AUC: 0.91; 95% CI: 0.89‐0.93) were as predictive as summary features (AUC: 0.90; 95% CI: 0.88‐0.92). Similar results were found when analyzing command and copy features separately.

**Conclusions:**

Functional features of the dCDT offer several advantages such as robustness to missing data, decreased subjectivity in feature selection, and avoidance of reducing complex information into summary statistics. In addition, there is potential to make inferences about cognition based on the shape and behavior of the functions. Further research is required to better understand what these functional features reveal about cognition.